# Seroprevalence of antibodies for bovine viral diarrhoea virus, Brucella abortus and Neospora caninum, and their roles in the incidence of abortion/foetal loss in dairy cattle herds in Nakuru District, Kenya

**DOI:** 10.1186/s12917-019-1842-8

**Published:** 2019-03-18

**Authors:** Tequiero Abuom Okumu, Njenga Munene John, James K. Wabacha, Victor Tsuma, John VanLeeuwen

**Affiliations:** 1 Department of Clinical Studies, Faculty of Veterinary Medicine, University of Nairobi, Nairobi, Kenya; 2 Zetech University, Nairobi, Kenya; 3 African Union Inter Africa Bureau of Animal Resources, Nairobi, Kenya; 4 Department of Health Management, Atlantic Veterinary College, University of Prince Edward Island, Charlottetown, Canada

**Keywords:** Abortion, Dairy cattle, *Neospora caninum*, Bovine viral Diarrhoea virus, *Brucella abortus*

## Abstract

**Background:**

No comprehensive studies have been carried out on the infectious causes of abortion in Kenyan dairy cattle herds. A survey was carried out to determine the seroprevalence of antibodies against Bovine Viral Diarrhoea Virus (BVDV), *Brucella abortus* (BA) and *Neospora caninum* (NC) among dairy cattle herds in Nakuru County, a major dairying area in Kenya. A prospective sero-epidemiological study was also undertaken to investigate the effects of BVDV, BA and NC on the occurrence of bovine abortion in dairy cattle herds, where monthly rectal palpations for pregnancy were performed, and monthly serum samples were tested for antibodies to the 3 pathogens.

**Results:**

In the 398 randomly selected cattle on 64 dairy herds, the seroprevalences of antibodies to BVDV, NC and BA were 79.1, 25.6 and 16.8%, respectively. Of the cattle seropositive to NC, 83.3% were also seropositive to BVDV and 13.7% to BA. Of the cattle seropositive to BVDV, 17.1% were also seropositive to BA. Among 260 monitored pregnant dairy cattle on the same 64 dairy farms, an incidence risk for abortion of 10.8% (28/260) was identified, while the incidence of other foetal losses was 1.1% (3/260). The incidence rates of sero-conversion for NC, BVD and BA were 1.1, 0.06 and 0.5 new infections/100 cow-months at risk, respectively. The foetal losses were mainly observed in animals less than 96 months old and occurred in mid-gestation. *Neospora caninum* was associated with most cases (29.0%) of foetal losses, followed by mixed infections of NC and BVDV (12.9%), BVDV (9.9%) and co-infections of BA and NC (6.5%).

**Conclusions:**

This is the first study to document the substantial incidence risk of BVDV and NC abortions in dairy cattle in Kenya, and demonstrates the relative importance of BA, BVDV and NC infections in dairy cattle in Kenya. Kenya laboratories should offer diagnostic tests for BVDV and NC to help farmers determine their roles in abortions on their farms. A comprehensive policy on the control of these important diseases should also be put in place by government with the involvement of all stakeholders in the dairy cattle industry.

## Background

Animal production systems are dependent on successful reproduction that leads to survival of a conceptus to subsequent reproduction [1]. While high fertilization rates of up to 90% have been reported, up to 65% of embryos are estimated to be lost before term, leading to significant economic losses and biological waste to the animal industry [2].

Some of the important infectious agents that have been reported to cause prenatal losses in cattle are Bovine Viral Diarrhoea Virus (BVDV), *Brucella abortus*, *Neospora caninum*, *Campylobacter foetus*, *Chlamydophila abortus*, *Escherichia coli*, infectious bovine rhinotracheitis virus, *Leptospira* spp., *Salmonella* spp., Rift Valley fever virus, and *Toxoplasma gondii,* as well as several fungal species, such as *Absidia* spp. and *Aspergillus* spp. [1, 3–8].

The diagnostic rate in bovine abortions is very low due to the diverse range of pathogens involved, as well as the fact that other factors affecting the dam, foetus and placenta may be involved [3]. Abortion also can follow an initial infection which may have gone on for several weeks or months; the aetiology of an abortion may not be detectable by the time the abortion occurs. The high cost of laboratory work for each pathogen also compounds the problem of under-diagnosis of abortions, with positive diagnostic rates of 17 and 43% having been reported in British and American dairy cattle herds, respectively [4, 5].

Reports on prenatal loss and pathogens that can cause abortion in Kenya are available; *Leptospira* and *Campylobacter* have been confirmed to occur [9–12]. A review of the records at Nakuru Regional Veterinary Investigation Laboratory (NVIL) revealed that between January 1997 and October 2007, 1182 cases of abortion were reported. Only 124 (10.4%) were positively identified as brucellosis while the rest (89.6%) had no definitive diagnosis. The other causes remained unknown, hence, interventions were difficult to institute to reduce the problem. Therefore, there is an urgent need for research to address causes of bovine abortion in Kenya and their associated risk factors.

Factors that have been reported to increase the risk of abortion in dairy cattle herds include: being a heifer; being an old cow (more than 10 years old); feeding on communal pastures; lack of vaccination against abortifacient diseases; and reproductive problems such as retained placentae, dystocia, uterine prolapse and stillbirth in the previous pregnancies [13, 14]. However, no studies have looked at risk factors of abortion from Bovine Viral Diarrhoea Virus, *Brucella abortus* and *Neospora caninum* simultaneously in a Kenyan context.

This study was designed to determine the seroprevalence of infections with BVDV, BA and NC, and the incidence risk of abortion/foetal loss caused by these 3 pathogens in dairy cattle herds in Kenya.

## Results

Of the 64 participating herds, grazing at 63.1% (251/398) was the most common method of rearing dairy cattle in this study, with the remainder being zero-grazed. The level of vaccination against reproductive diseases in dairy cattle selected for this study was low. Only 7 (1.8%) and 4 (1.0%) cattle had been vaccinated against brucellosis and BVD, respectively. All these vaccinations had been done in animals at one farm.

For the 398 cattle in the survey, 242 (60.8%) were from small-scale farms, while 156 (39.2%) were from large-scale farms. Friesians were the most common breed encountered, comprising 68.1% (271) of the selected animals. The rest of the breeds were Ayrshire at 18.1% (72/398), Guernsey at 6.0% (24/398), Jersey at 5.5% (22/398), and Sahiwal at 2.0% (8/398), and 1 cow (0.25%) was a crossbred cow of unclear breed.

About age, 178 (44.7%) were 49–96 months old, 132 (33.2%) were 13–48 months old, 84 (21.1%) were more than 96 months old and 4 (1.0%) were between 6 and 12 months old. The parity of the selected animals ranged from 0 to 9. The mean parity was 3.1 with a standard deviation of 2.1, and median of 3. Fifty-seven (14.3%) animals had not calved before.

Regarding breeding, 280 (70.4%) cattle were bred by artificial insemination using imported semen, 85 (21.4%) were bred by artificial insemination using local semen, and only 1 (0.4%) had received embryo transfer. Of the 398 animals sampled for the prevalence survey, 32 (8.0%) had never been bred yet.

Among the 398 cattle that were included in the prevalence study, BVDV had the highest seroprevalence at 79.1% (95% CI = 75.2–83.0%). The seroprevalence of NC was 26.0% (95% CI = 21.6–29.6%) while that of BA was 16.8% (95% CI = 13.2–20.4%).

Eighty-five (83.3%) of the 102 dairy cattle that were seropositive to NC were also seropositive to BVDV, and 14 (13.7%) were also seropositive to BA. In addition, of the 315 dairy cattle seropositive to BVDV, 54 (17.1%) were also seropositive to BA*.*

In the prospective study, of the 260 animals monitored, 31 (11.9%) experienced reproductive wastage; the incidence of abortion was 10.8% (28/260), while the incidence of early embryonic death, deformed foetus at term and foetal mummification was 0.4% (1/260) for each condition.

Of the three infections under investigation as causes of abortion/foetal loss, a solo infection with NC was associated with the most foetal losses at 29.0% (9/31), followed by mixed infections with NC and BVDV at 12.9% (4/31). Table 1 shows other combinations of infections, including BA and NC mixed infections which led to 6.5% of the foetal losses (2/31). Ten (32.3%) of the 31 cases of foetal loss were not associated with any of the three infections under investigation. The animal that had foetal mummification had a four-fold increase in antibody titres to BVDV, while the animal that had a deformed foetus had a four-fold increase in antibody titres to NC and BVDV. The single case of EED was not associated with any infection.

**Table 1:** Distribution of infections associated with foetal loss (*n* = 31) in 260 dairy cattle in Nakuru District, Kenya, 2010–2011

Abortifacient pathogen	Number	Percentage
NC	9	29.0
NC and BVDV^1^	4	12.9
BVDV	3	9.7
BA	2	6.5
NC and BA^1^	2	6.5
NC, BA and BVDV^1^	1	3.2
No NC, BA or BVDV infection	10	32.3
Total	31	100.0

^1^Indicates animals that had co-infections (i.e four-fold rise in antibody titres to more than one pathogen with subsequent foetal loss)

Eighty percent of the foetal losses occurred between 4 and 7 months of gestation. The gestation details are shown in Table 2.

**Table 2:** Stage of gestation of foetal loss (*n* = 31) in 260 dairy cattle in Nakuru District, Kenya in 2010–2011

Month	Number	Percentage
< 2	1 (E.E.D.)	3.2
3	2	6.5
4	7	22.6
5	5	16.1
6	8	25.8
7	5	16.1
8	2	6.5
Term	1 foetus with deformities	3.2

On the unconditional regression analyses, factors significantly associated with the occurrence of foetal loss were: being of young age (*P* = 0.02, OR = 1.4), Friesian breed (*P* = 0.04, OR = 1.93), parity< 3 (*P* = 0.01, OR = 2.9), and being bred using artificial insemination with imported semen (*P* = 0.05, OR = 2.1). In the results of the multivariable regression analysis, no models contained more than one significant variable.

## Discussion

This is the first study to document the substantial incidence risk of foetal loss due to BVDV and NC in dairy cattle in Kenya, and demonstrates the relative importance of BA, BVDV and NC infections in dairy cattle in Kenya. Kenyan veterinary laboratories should offer diagnostic tests for BVDV and NC to help farmers determine their roles in abortions on their farms.

In this study, all three pathogens were associated with substantial reproductive wastage. While BVDV was the most common abortifacient pathogen in this study population, NC was associated with the most foetal losses (29.0% by itself). NC has been shown to be one of the most common causes of foetal loss elsewhere, responsible for 3.9–69% of all diagnosed abortions in dairy cattle herds [5, 15–29]. Most BVDV abortions occur when previously unexposed dams are infected during gestation [30, 31]. Thus, with the high prevalence rates of BVDV in this study, most BVDV-infected animals may have been previously exposed and recovered, leading to the low rates of abortion/foetal loss attributable to this pathogen. Previous studies have tried to investigate interrelationship between BVDV and NC as causes of abortion/foetal loss [32]. In our study, co-infection by BVDV and NC led to 12.9% of the foetal losses.

Positive diagnostic rates for foetal loss of 67.7% were achieved. This was much higher than rates of between 17 and 56.3% that have been reported in previous studies [4, 5, 29]. This high rate of positive diagnosis may have been because the animals selected into the study were closely monitored until the foetal loss occurred. Samples had therefore been collected prior to the foetal loss and after, thus increasing the chances of diagnosis. All the other reports were based on samples collected after the foetal loss. By this time, it is not easy to detect the aetiology since infection normally precedes foetal loss by weeks or months [4, 5].

Several infections, such as BA and BVDV, have been reported to be transmitted through the use of artificial insemination [33]. In this study, the frequency of abortions was significantly higher in dairy cattle bred by artificial insemination using imported semen relative to those AI bred using local semen. Most of the animals bred using imported semen were on breeder farms who had high traffic of cattle buyers/traders particularly in-calf heifers. In addition, the poor biosecurity measures seen on most farms in this study may have led to increased chances of transmission of some of these pathogens, such as BVDV, by animal health providers, as has been reported in previous studies [34].

*Neospora caninum* was the pathogen most associated with foetal loss in our study. Abortions in cattle due to NC have been reported to occur commonly from 5 to 6 months of gestation [6]; this may be the reason why 80% of the abortions/foetal loss in this study were recorded during the period from 4 to 7 months of gestation. Cases of foetal loss were also more common in young and middle-aged dairy cattle. Indeed, no dairy cattle more than 96 months old (*n* = 84) had abortion or any foetal loss, which may be because younger animals are naive to most abortifacient pathogens, thus making them more likely to contract these infections and subsequently abort. In addition, NC was involved in nearly 50% of the abortions, and its vertical transmission often leads to abortions in younger cattle [30]. In fact, the age of the dam was the only statistically significant risk factor to the occurrence of bovine abortion in this study, although with only 31 cases of foetal loss, the power to detect significance of the differences was limited.

The main limitations of our study were the failure to recover aborted foetuses as well as placental tissue, which would have helped to enhance the diagnosis of the actual causes of abortion. Farmers and animal health providers should be informed on the importance of submitting these samples in cases of abortion.

Further studies should be carried out to assess whether the abortifacient pathogens herein studied influence the milk production in dairy cows from Kenya as well as their economic impact in the dairy cattle industry of this country. Other abortifacient pathogens in cattle could also be investigated, such as leptospirosis and infectious bovine rhinotracheitis virus.

## Conclusions

We found a substantial incidence risk of BVDV and NC abortions in dairy cattle in Kenya. The seroprevalences of BA, BVDV and NC infections were relatively high in the tested dairy cattle in Kenya, especially BVDV and NC. Kenya laboratories should offer diagnostic tests for BVDV and NC to help farmers determine their roles in abortions on their farms. A comprehensive policy on the control of these important diseases should also be put in place by government with the involvement of all stakeholders in the dairy cattle industry.

## Methods

This study was carried out in Nakuru County, Kenya. It is one of the main dairy farming zones in Kenya and is also the main catchment area for dairy cattle breeding stock in Kenya and the East African region. The dairy cattle population in this area ranges from 100,000-120,000, most of which are large herds on large-scale farms. Though Nakuru is a large-scale farming district in terms of land area, with many large-scale farms averaging 1100 acres, many small-scale farms with average sizes of between 0.3–10 acres do exist [31–33].

### Study farms and animals and data collection for the survey

This phase of the study was carried out between January and May 2010. A list of more than 300 dairy cattle farms was collected from the local animal production office and dairy societies (the sampling frame), and divided into small- and large-scale farms, with large-scale farms having > 30 dairy cattle and small-scale farms having ≤29 cattle. A stratified random sampling procedure was used: 50 farms having < 29 dairy cattle and 20 farms having > 30 dairy cattle were randomly selected into the study. Herd sample size was determined based on the number of farms that could logistically and financially be handled for the study. Of the 70 farms, 64 agreed to participate.

For the prevalence survey, blood samples were collected from 398 randomly selected dairy cattle (pregnant and non-pregnant; over 6 months old) of the estimated 200,000 dairy cattle on the participating farms, based on the following formula

$$ n=\frac{4\times \mathrm{P}\left(1-\mathrm{P}\right)}{L^2} $$

where: n = sample size, L = Precision (0.15) and P = incidence risk estimate (25%), using 95% confidence levels [35].

These selected cattle were restrained in a crush, and the ventral aspect of the tail disinfected with ethyl alcohol-soaked cotton swabs. Vacutainer needles (BD Vacutainer® blood collection needle) and serum tubes (BD Vacutainer® blood collection tubes) with clot activator were used to collect the blood samples. The blood samples were centrifuged at 2500 rotations per minute for 15 min, and serum was separated and frozen at -20 °C until all samples were collected, at which time, laboratory tests were conducted.

Data collection on the participating farms and cattle was conducted through a face-to-face interview with the farm owner or manager, obtaining information on cattle age, breed, parity, and breeding at the cow level, and at grazing and vaccination practices at the herd level.

### Study farms and animals and data collection for the prospective study

This phase of the study was carried out between January 2010 and May 2011. On monthly visits to the same 64 farms, 279 dairy cattle that were confirmed pregnant by rectal palpation (40–60 days post service) were selected for the prospective study. However, 19 dairy cattle were lost to follow-up due to sales of the animals from the farms, leaving 260 pregnant cows for the analyses.

Rectal palpation was performed monthly to test for continued pregnancy in cows, and blood samples were collected monthly until the cow calved or the pregnancy was lost (abortion/EED/mummification). The time of pregnancy loss was when the foetal loss was detected, by the veterinarian through rectal palpations and/or by farmers finding a foetus or vulvar foetal membranes, and the date of an abortion was estimated retrospectively.

Again, blood samples were centrifuged, and serum was separated and frozen at -20 °C until all samples were collected, at which time, laboratory tests were conducted. Data collection on the participating cows was again conducted through a face-to-face interview with the farm owner or manager.

### Laboratory analyses

For the survey, commercial Enzyme-Linked Immunosorbent Assays (ELISA) were used to screen for antibodies against bovine viral diarrhoea virus, *Neospora caninum* and *Brucella abortus* antibodies (IDEXX Laboratories, Switzerland AG). For the prospective study, the same blood testing was done monthly to monitor changes in antibody titres (to BVDV, *Brucella abortus* and *Neospora caninum*). This method of analysis was selected due to its high sensitivity and specifity as well as its ability to test large numbers of samples at one time. The intra- and inter-test variability was minimized by using the same laboratory and technologist to perform all the testing.

### Statistical analysis

Data from the serological survey and prospective study were entered and stored in Microsoft Office Excel 2007 (Microsoft Corporation, 2007). The data were imported into Genstat® 13th edition, service pack two, for analysis (VSN international).

Descriptive statistics, including prevalence and incidence risk, were computed for the serological survey and abortion parameters. To calculate cause-specific incidence risks of abortion, monthly antibody levels to BVDV, BA and NC were examined from samples before and after the reported abortions, with a four-fold increase in a titre for a specific pathogen indicating the likely aetiology of the abortion by that pathogen [36].

While the purpose of the study was not to identify risk factors to foetal loss, we did conduct Pearson’s Chi-square tests to determine significant differences in dichotomous predictor variables between outcome groups (e.g. those that aborted versus those that didn’t abort). Multivariable logistic regression was carried out to model the incidence risk of abortion in dairy cattle in Nakuru County, and odds ratios, as a measure of strength of association between the significant model variables (*P* < 0.05) and the outcome, foetal loss, were calculated. The backward elimination procedure was used for regression, and factors that were significant (P < 0.05) were retained in the final model. Potential clustering of animals within farms was controlled for by including farm as a random effect in the modelling.

## Abbreviations

BA: *Brucella abortus*
BVDV: Bovine Viral Diarrhoea Virus
EED: Early Embryonic Death
ELISA: Enzyme-Linked Immunosorbent Assays
NC: Neospora caninum
NDDP: Nakuru District Development Plan
NVIL: Nakuru Regional Veterinary Investigation Laboratory
OR: Odds ratio
